# Implementation of drone based delivery of medical supplies in North-East India: experiences, challenges and adopted strategies

**DOI:** 10.3389/fpubh.2023.1128886

**Published:** 2023-06-02

**Authors:** Sumit Aggarwal, Prakamya Gupta, Nupur Mahajan, Sivaraman Balaji, Khangembam Jitenkumar Singh, Balram Bhargava, Samiran Panda

**Affiliations:** ^1^Indian Council of Medical Research (ICMR), New Delhi, India; ^2^National Institute of Medical Statistics (ICMR), New Delhi, India; ^3^Department of Health Research, Ministry of Health and Family Welfare, New Delhi, India

**Keywords:** healthcare delivery, public health, difficult terrains, India, drone

## Abstract

Timely delivery of medical supplies is essential in the healthcare sector, which is hampered by factors such as poor transportation network, traffic and adverse environmental conditions. Alternatively, drone operations can leapfrog the last mile logistic solutions in hard-to-reach terrains. The present paper elucidates the implementation process of drone-based delivery of medical supplies, operational challenges and innovations adopted by scientists in Manipur and Nagaland. Three districts, Bishnupur, Imphal West and Churachandpur from Manipur and two districts, Mokokchung and Tuensang from Nagaland, were selected for the study. Regulatory and ethical approvals and coordination with state health and administrative authorities were accorded. Implementation and operational challenges faced by the research team were recorded elaborately in the field diaries and assessed qualitatively. The experiences encountered by the team for case-to-case based permission and coordination with the central and state aviation authorities, district administration and health authorities were observed. The drone-related technical and logistic challenges were identified as the deployment of suitable drones, payload capacity, time management for operations, and transportation of drones. The officials adopted mitigation strategies to overcome field-based challenges. Drone-based deliveries of medical supplies are proving to be time efficient, however, overcoming operational challenges could provide an effective long-term deployment strategy.

## 1. Introduction

Unmanned Aerial Vehicles (UAVs) or drones are emerging technologies with the potential to leapfrog the last mile logistics solution for transporting medical supplies thus, strengthening the healthcare system. Furthermore, this transportation technology is being explored in military, agricultural and food sectors for logistic support (1). Additionally, drones have been used extensively in search and rescue along with delivery of medical aids and food packages during calamities in Nepal, Haiti, Caribbean islands (2). In Papua New Guinea islands, feasibility trials were conducted where dummy tuberculosis samples were transported from remote locations to high-resource diagnostic centres via drones (3). This was a significant intervention as the country was experiencing high burden of tuberculosis with increased multidrug resistance. In the third world countries such as Malawi, Rwanda, etc. drones have been used for delivering HIV testing kits, contraceptives in rural areas which immensely reduced the time for testing new born infants of mothers with HIV (4). Blood bag and critical care medicine delivery in inaccessible and remote locations from hospitals have also been in practice in Rwanda through drones (2). In India, the northeast region is well-known for its difficult and inaccessible terrains compared to other geographical parts of the country. Moreover, the service deliveries through drones in the North-Eastern states of India remain unexplored, and this study may ensure a boost in the possibility for the last mile deliveries efficiently in the future.

The major criteria for a better healthcare system are universal and adequate access, affordability, accountability and empathy of service providers, quality care and the cost-effective use of resources, and wide coverage and attention to vulnerable groups (5). To ensure this, India has a three-tiered healthcare system that provides preventive and curative healthcare services to the rural and urban regions (6). Also, in India, with a gradual push towards enhancement and improvisation of the healthcare sector, advanced technology solutions offer an expedited path towards accessibility of resources (7). The advent of COVID-19 pandemic and the restrictions imposed due to lockdowns provided opportunities for flourishing alternative methods supporting contactless delivery of services in varied destinations (8).

Despite significant cited advantages of the use of drones in healthcare, there has been little scientific documentation about the practicality, acceptability, operational challenges and impact on the physical characteristics of the medical supplies (9). On the other hand, pioneering feasibility studies for integrating novel technologies in the existing healthcare system have several challenges at the operational level experienced by the implementing unit and perceived challenges among the stakeholders, frontline workers and communities who will be receiving the benefits of the technology, at large (10). Thus, the present study documents the operational challenges experienced by the study team while conducting the feasibility study for drone-based delivery of medical supplies. Additionally, the paper also provides the tested solutions for the operational challenges encountered to aid an efficient operation. This study was conducted in diverse geographical terrains of Manipur and Nagaland including islands, foothills, valleys, and mountains with varying climatic conditions by use of variety of lightweight drones.

## 2. Theoretical foundation

Integrating drone technology with the existing conventional mode may help in overcoming the susceptibility to human errors and relatively higher time-consumption faced in the later (11). Amidst the economic crisis evidenced with losses in jobs and business, the upcoming surge in requirements of drones is creating a promising space for boosting the economy with increase in drone industries globally (12, 13). There are several theoretical foundations employed in the introduction of technological advancements to explore its acceptance with regards to drones (13). One such conceptual foundation is the risk theory which investigates the public acceptance and relies on perceived risk as the sole indicator (14, 15). Thus, this theory forms the theoretical foundation for the feasibility study of drone-based healthcare deliveries in North-Eastern states of India to assess the acceptability and adaptability of this technology among the stakeholders, healthcare workers and communities in the study area. This article is a sub-set of the main study, and documents the challenges of the team engaged in the implementation in the drone operations in real-time field situations. Thus, the risk theory forms a pivotal foundation for understanding these challenges which can be further envisaged by the drone operators for effective deployment strategies in similar operations.

## 3. Materials and methods

The present Beyond the Visual Line of Sight (BVLOS) study was planned to test the feasibility of drones to transport medical supplies in geographically difficult terrains of North-Eastern states of India. In total, five districts were selected, i.e., three from Manipur (Imphal West, Bishnupur and Churachandpur) and two from Nagaland (Mokokchung and Tuensang) considering the diverse geographical terrains. For instance, the districts of Manipur have geographical landscapes consisting of foothills, islands, valleys and flatlands, and the districts of Nagaland are mainly covered with dense forest and mountainous terrains. Among these five districts, 19 healthcare centres were chosen for the study.

### 3.1. Study approvals

**Figure 1:**
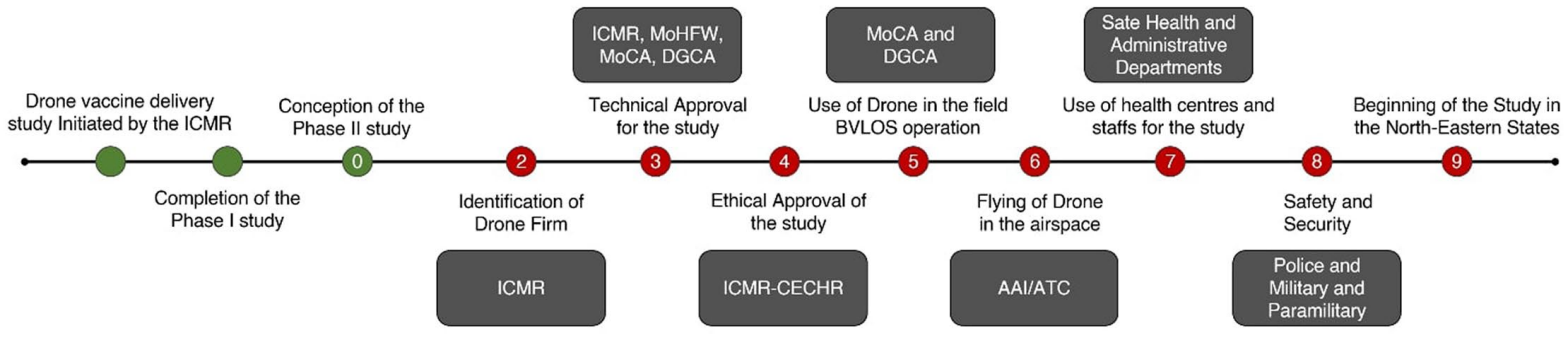
Flowchart of approvals sought from various authorities.

Regulatory, technical and administrative approvals were obtained from concerned authorities such as the Ministry of Civil Aviation (MoCA), Government of India, Director General of Civil Aviation (DGCA), Airport Authority of India (AAI), and Air Traffic Control (ATC), respectively. The approval on the Standard Operating Procedures (SOPs) for Drone operation was obtained from the DGCA. Since the drones were permitted to fly up to 400 feet altitude Above Ground Level (AGL) in Beyond the Visual Line of Sight (BVLOS), approvals from the AAI and ATC were also sought. The local flight route planning and timings were coordinated with the civilian ATC and defence ATC on real-time basis. The state administrative and health authorities were contacted for supporting in the identification of sites and coordination with local authorities for awareness among the general public, safety and security concerns. All the pre-requisite essential approvals were also obtained from locally present military and paramilitary forces (Figure 1). In addition to these regulatory approvals, technical and ethical approval for conducting the study was obtained from the Technical Expert Committee and Institutional Ethics Committee (Ref No. CECHR-007/2021), respectively.

### 3.2. Study implementation and data collection plan

As per the Drone Rules 2021 (14), the health centres including district vaccine and drug storage departments (DVDs), district hospitals (DH), community health centres (CHC), and primary health care centres (PHC) falling under the green zone (above 12 km from the airport perimeter) were chosen for study. Subsequently, the approvals were sought on developed flight route plans from the district health authorities and local ATC. For safety and security purposes, the flight maps and schedules were reported to the police department and military troops in the operational sites. An interdisciplinary team consisting of members from medical, anthropology and technical backgrounds were monitoring, supervising, and documenting the execution of all the activities. Participatory observations were documented by each team member as they were thoroughly involved in the entire process of medical supply delivery at different stages. Each team member maintained a daily field diary for recording their observations, experiences and challenges faced during the implementation of the activities at ground level. The team conducted discussions every night after finishing the field operation hours to pave ways to overcome the difficulties faced. Thus, the present research article highlights the experiences of the research team through participant observation. It is envisaged that overcoming these challenges will enable the development of an ecosystem for efficient drone-based medical supplies delivery in India.

## 4. Results

**Figure 2:**
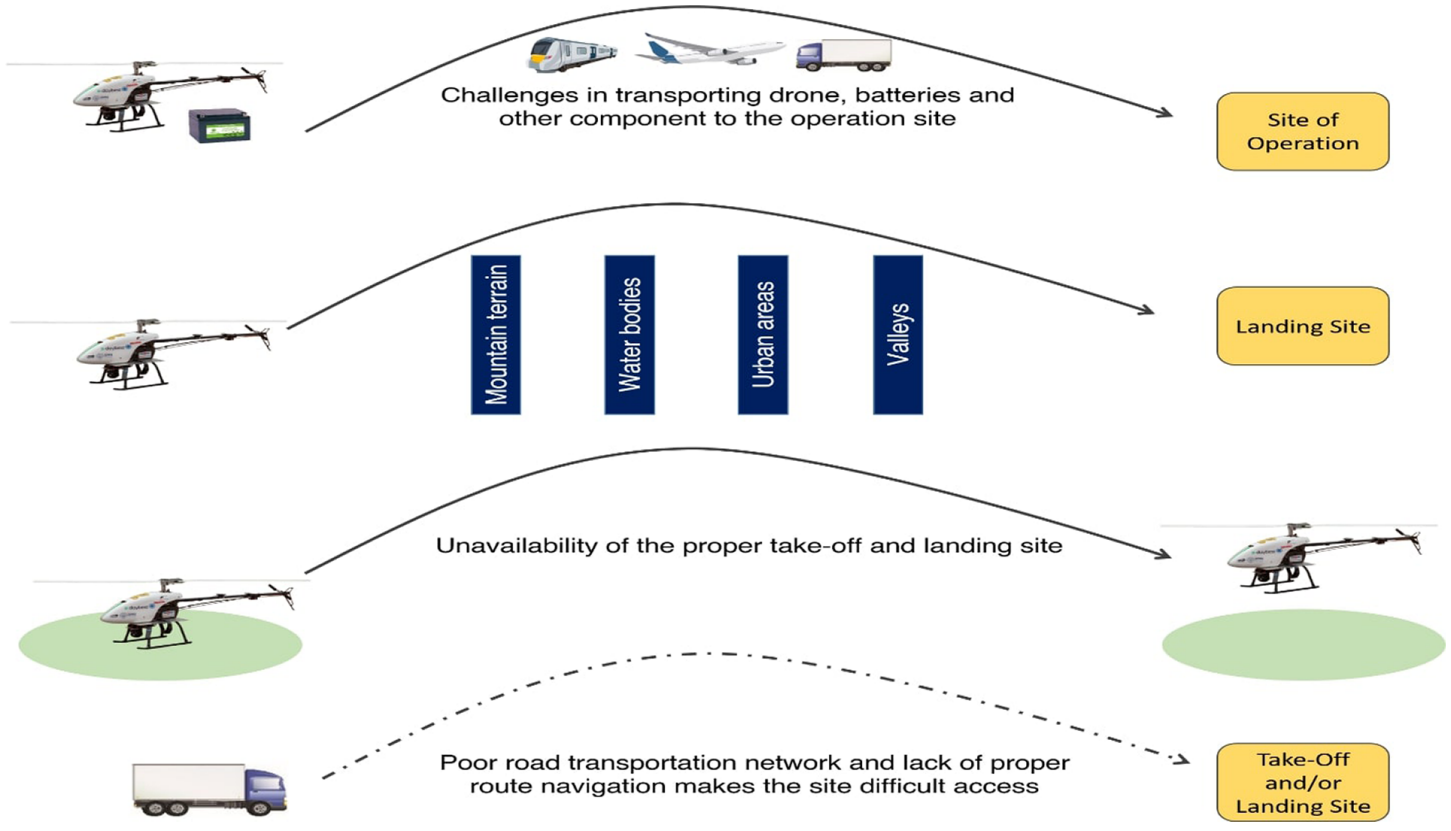
Various challenges faced during the field operations in Nagaland and Manipur.

The present study was a unique and pioneering initiative in South Asia which delivered about 20,000 units of medical supplies to various hard-to-reach terrains of North-East India. The new drone rules (2021) provided flexibility for conducting BVLOS operations under 400 feet AGL in green (above 12 km from the airport perimeter) and yellow zones (above 5 km from the airport perimeter) across India, thus, ushering in growth while balancing safety and security (16). With the inception of the study, the team faced a few challenges in obtaining technical permission, ethics approval and flying rules approvals. Additionally, field-level operational challenges were also experienced by the officials. The major challenges encountered by the field team are illustrated in Figure 2 and discussed in detail in the following sections.

### 4.1. Obtaining regulatory approvals for drone operations

#### 4.1.1. Coordination with the central and state aviation authorities

According to the Drone rules 2021, drones are allowed to fly without any additional permissions, except in red zones (16). Under this provision, MoCA granted us the permission for conducting the study in the selected locations that falls under yellow and green zone. For this, identified sites, where the drone’s flying pathway passes through the red zone as per the original plan were modified. Additionally, the concerns related to the proximity of the flying sites to the international borders were priorly informed to the army ATC for information and coordination.

#### 4.1.2. Procurement of unique identification number and keyhole markup language files from drone operators for issuance of notice to airmen (NOTAM)

In India, all UAVs must register on the DigitalSky platform and obtain a UIN. Therefore, UIN numbers were obtained by the drone firm before deployment of drones at the study sites. Subsequently, the NOTAM was obtained from the AAI, which are notices containing information concerning the establishment, condition or change in any aeronautical facility, service, procedure or hazard distributed via telecommunication for the timely dissemination of drone flight related knowledge to personnel concerned with flight operations (17). In addition, the UAV operations must be confined to pre-determined routes defined by waypoints and committed to lateral and vertical confinement. In this regard, KML waypoints duly plotted on Google Earth need to be shared with the AAI and ATC in advance for the screening of the route and approval. Thus, the KML files and a tentative flying schedule were submitted to AAI to obtain the NOTAM to alert pilots flying in the vicinity of the flying areas on the days of drone operations. All nearby civil and defence ATC were informed regarding the NOTAM as well.

#### 4.1.3. Coordination with local ATC

Along with the AAI approvals, coordination with the local airport and ATC is necessary for safe drone operations. For this, real time permissions were obtained from the regional and defence ATC of the study sites. Information on the ‘No Drone Zone’ or the ‘Red Zone’ was also provided by the regional ATC. However, the drone operations were restricted to 400 ft. AGL and conducted during watch hours, i.e., between 8.15 AM to 5 PM of the Imphal and Manipur ATC. In addition, few air spaces of our study were under the control of defence forces, i.e., ATC-Jorhat, Assam where, drone operations were restricted due to the movement of defence or private helicopters. Our team assured the ATCs that Remotely Piloted Aircraft Systems (RPAS) operations will be terminated immediately in event of emergency/VIP movement or unfavourable situations demanding the safety of flight operations. For instance, one of our drone operations were temporarily suspended when instructions were received from ATC, Imphal during the extremists’ attack on the convoy of the Indian army in Churachandpur, Manipur (18). This in turn affected that particular operation and subsequent plans for the emergency delivery of medical supplies at other sites as well.

#### 4.1.4. Concerns of the ethics committee regarding the safety of stakeholders

The concept of drone-based delivery of medical supplies raised several ethical concerns such as emergency landing or loss of drones, injury to the person handling the drone, unknown attack on the drone, crashing of drones in the residential area, managing and transporting live vaccine or toxic material, damage to the biological material, insurance coverage in case of emergency, etc. The study investigators provided detailed explanations to the committee regarding development of training modules and provision of hands-on training to drone operators and health care workers, utilisation of seal-proof containers to carry vaccines and other supplies, accidental insurance of drones related damages and the incentives covered under it, strategizing route mapping away from densely populated areas and availability of the emergency medical team. Based on these clarifications, the Institutional Ethics Committee (IEC) granted ethical approvals for the study in Manipur and Nagaland. In addition, owing to the diversity in the cultures, customs and languages in the north-eastern parts of India, the local community-based organisations were also involved in operations for local understanding and coordination.

### 4.2. Coordination with the local authorities

#### 4.2.1. Support and coordination from state health authorities

Transportation of medical supplies from district storage to peripheral health care centres like Community Health Centres (CHCs) and Primary Health Centre (PHC) requires close coordination to the district health authorities (DHA), which regulates the distribution of medical supplies among the district. Generally, on the request of a medicine indent from PHC or CHC, DHA releases the medicines from the district storage facility to the concerned health centres. After understanding this mechanism of supply chain and continuous discussion with the state and district health officials including state mission director, state and district immunization officer, district vaccine managers and medical officers, the study team identified the possible take-off and landing sites near the medical supply depot and PHC or CHC, respectively, to minimise the transportation time from the stock site to the take-off/landing areas.

#### 4.2.2. Coordination with security agencies

Since few study sites were located closer to international borders and army camps deemed as sensitive/ insurgency prone areas, the coordination from local police, army/para-military regiment and intelligence personnels was requested for safe and secured drone operations. For instance, in Manipur, the take-off point of one site was in close vicinity to the army regiment, which was a ‘No Drone Zone. Since the study team was unaware regarding their settlement, the army was not informed beforehand. However, on the first day of trial the army officials enquired about the project and the study team present their informed the senior army officials regarding the study and provided all the relevant documents and permissions. Thereafter, the study team was continuously communicating with the local police, intelligence bureau officer and paramilitary forces about the drone operation on real-time basis.

### 4.3. Technical challenges related to drones in the field setting

#### 4.3.1. Drone selection

According to the Drone Rules 2021, drones have been classified into nano (<250 gm), micro (>250 gm but <2 kg), small (>2 kg but <25 kg), medium (>25 kg but <150 kg) and large drones (>150 kg) based on the overall their weight. In the present study, various types of small drones such as fixed-wing, multirotor, single-rotor helicopters, and fixed-wing hybrid vertical take-off and landing (VTOL) were used according to the payload, landscape, weather, etc. In general, different drone types have varied merits and demerits, thus, the type of drone was selected as per the operations (Table 1). For instance, a single rotor helicopter or a hybrid VTOL were used for carrying more payload for a longer distance and on mountainous terrains. Whereas, a quadcopter was suitable for delivering a payload of <1 kg within the range of 5 km–10 km. Although a fixed-wing has advantages over Quadcopter/Hexacopter drone types in terms of payload capacity and data-link equipment, but due to the runway requirement for landing and take-off, that type was not used in areas with smaller spaces. In this study, choosing the drone in terms of the payload capacity and distance to be covered was challenging thus in-depth discussions were conducted with the drone operators and state health department in this regard. Since the quality and quantity of the medical supply delivery may vary in a case-to-case manner, the type of drone was selected accordingly for each location.

**Table 1:** Types of drones, their key features and advantages.

S. No	Type of drone	Key Features	Suitability
1	Multirotor Drones: Quadcopter/ Hexacopter	Lightweight, less complicated operations, ease of use, handy in nature, easy assembly.	Short distances, (total up to 10–12 km) Small landing or take off-site, quick operations as less preparation time required used in Nagaland: Mokokchung DH to Ungma PHC (6 km)
2	Single rotor helicopter	More stable while flying, and speedy, it can drop payload and return to its original site, suitable for mountain ranges	Moderate distances (25–32 km) In an emergency it can go drop the payload and return to the original site, can carry vaccines, medicines, the advantage of true VTOL escalation and descent, Useful in steep hilly region used in operation from Bishnupur DH to PHC Sekmaijin (32 km)
3	Fixed-wing Hybrid VTOL	More stable while flying, and speedy, it can drop payload and return to its original site, suitable for all type of landscapes	Long distances (60–120 km), More endurance, suitable for flatlands as well as the hilly region With more carrying capacity, intra-district movements are convenient operations were conducted with this drone from Mokokchung DH to Tuensang DH (80 km)

#### 4.3.2. Payload capacity

Owing to variety of medical supplies such as vaccines, tablets, syringes, glass bottles, PPE kits, gloves, etc. and requirements from the health centres, each drone operation required different payload capacity. Thus, the dimension of the carrier box was determined based on the type of medical supplies so that the payload capacity may be efficiently used. However, it must be carefully examined that the consignment may not exceed the maximum payload capacity of the drone. As per the field experience, if payload chambers are customisable, they can be fixed to the drone for delivering the medical supplies.

#### 4.3.3. Placing the conventional carrier box and maintaining optimal temperature in the drone

Temperature-sensitive materials such as vaccines, serums, etc., must be transferred in a controlled environmental condition to maintain the temperature as per the manufacturer’s requirements. In this regard, the CB used in the Universal Immunization Program (UIP) has a dimension of 25×25×30 cm and an empty weight of 2.30 kg, which is heavier and bigger in size, therefore, not suitable for drone transportations. For instance, if the indent for the vaccine is less, i.e., 10 vials and the aerial distance to be covered is less than 10 km, the quadcopter drones with the capacity of <2 kg is sufficient for the delivery. However, the conventional CB used for this purpose other high-capacity drones are required which will not cost effective. To overcome this issue, smaller carrier boxes with an empty weight of 0.5 kg were used in the present operations, which could maintain the required temperature (2°C to 8°C). A few dry sorties were conducted without vaccines or any medical supplies to assess the capacity of the CB to maintain the inner temperature. For this, an Electronic Data Logger Monitor (EDLM) was placed along with ice packs and vaccines to monitor the temperature. Additionally, for delivery of non-temperature sensitive supplies soft boxes were used, which were 100 grams in weight. Therefore, the CB that could maintain the lower temperature (2°C to 8°C) may be customised according to the drone type and the quantity of the medical supplies.

#### 4.3.4. Unavailability of advanced machinery parts/sensors of drones

Several sensitive drone components such as Avionics- Autopilot, Remote Controller, Radio Frequency Module, high-capacity batteries etc., are not manufactured in India. The current policies require an industrial licence for importing these components from abroad, therefore, their non-availability is a major concern as the import process is time-consuming and expensive. This badly impact the research and logistics of various drones in India. For instance, altitude sensing is an important feature for any drone to maintain the flight manoeuvres like landing, taking off, maintaining AGL, etc. The existing *Li*ght *D*etection *a*nd *Ranging* (LiDAR) sensors measure the distance between the sensor and objects in their field of view (19). However, these sensors operate at ~30 frames per second (fps), whereas the drone flies at ~20–25 m/s leading to a delay of a few milliseconds in accurately detecting the object in its path. As drone operations have been restricted to 400 ft. AGL in India (irrespective of the zone classification) limited resolution and slow scanning rates make it impossible for drones to distinguish and maintain the AGL limits, especially while crossing a hilly terrain. Therefore, advanced LiDAR systems along with computer vision and artificial intelligence navigation systems is needed for path prediction, object classification, and threat assessment, for maintaining a constant AGL altitude. Moreover, emergency operations at night require these sensors to be installed within the drone for medical supply delivery. Thus, either the policies should ease the importing of these sensors or the manufacturing process should be initiated for the effective drone operations not only in the healthcare sector but, in agriculture, logistics sector as well.

#### 4.3.5. Use of high voltage batteries and their transportation to the implementation site

Batteries are the vital part of drones providing power to every component on board, which varies as per the drone type. A suitable set of batteries for a drone is determined by comparing the power, energy, weight, cost, safety and maintenance (20). Although voltage and capacity are important, weight and discharge rate (also called a ‘C’ rating) should also be considered for drones that transport medical supplies. Because, the discharging of batteries in the middle of the flight can cause forced landing in unplanned areas where recovery of the drone may be challenging and leakage of medical supplies may cause ethical concerns. For instance, the use of drones in hilly terrain would require climbing high-altitudes which will consume high voltage of batteries in drones. For this, high-capacity lithium polymer batteries (22000–32000 mAh, 22.2 V, 6C) are required which will enhance the flight time and drone performance, however, the flying distance will be reduced. In addition, the availability of charging stations at the take-off and landing sites adds another challenge for the UAV operator. In the present study, double battery-operated drones were used to increase the endurance and capacity of the batteries. Additionally, with the help of army officials, a makeshift extension of the charging point was set up at the take-off site for charging the extra set of batteries, so that they can be used interchangeably. This improved the time efficiency of the operations as more sorties could be conducted in a single day. However, still there is a need for low weight and high-capacity batteries for transporting medical supplies to a longer distance.

#### 4.3.6. Loss of telemetry data during the flight

For BVLOS flights, real-time information and communication with drones are essential to know their location in the airspace. However, the loss of telemetry data during the flight remains a major challenge for BVLOS operations where the drone is remotely monitored by the operator from the command centre. For instance, in our study, few operations were conducted in dense forest and mountain ranges of Manipur and Nagaland, where GPS network connectivity was poor at some points, which affected the monitoring of the drone path. Thus, additional GPS drone tracking device, i.e., advanced radio frequency (RF) modules were used to get undisturbed connectivity during the drone flight. This technology also enabled communication and navigation systems to operate autonomously or semi-autonomously up to 5 kilometres. Alternatively, cellular or GPS-based devices were installed in the drone to have better information about its location. By installing the high-capacity path detecting technology these issues can be resolved especially in the remote areas.

#### 4.3.7. Skilled manpower for flying drones

Highly skilled and trained drone pilots and manpower are required for any field operations especially for critically plotting the waypoints, preparing the flight plans, maintenance of the aircraft, monitoring of take-off and landing, etc. In the healthcare delivery, the drone personnels are required at take-off and landing points to send and receive the payload, which requires an additional manpower moving to the landing site every time. In the present study, the drones were programmed for automatic landing after which the payload got detached from a hook and clip mechanism following an automatic take-off. In this way, after the drone took off from the landing site, the box was picked up by the healthcare worker, which did not require any additional technical staff. Moreover, a single pilot at the command centre was sufficient to manage the whole operation. This kind of deployment may be useful in the long run to adopt drone-based delivery models in healthcare as limited human resource requirements will make the operations more cost and time effective.

#### 4.3.8. Safe transportation and storage of drones

After the completion of operation for the day, the safe storage and security of the drones near the deployment site remain a major concern, considering their high maintenance cost, chances of misuse, damage causing delays in operations. In the present study, the drone was safely kept at the District Health Authorities office in Manipur as well as in Nagaland.

#### 4.3.9. Registration and third-party insurance of the drone

BVLOS operations carry significant safety concerns ranging from injured bystanders to damage or trespassing of personal property to invasion of privacy. According to Drone Rules-2021 (16), the drone cannot operate in India without a valid third-party insurance policy. At present, there are a handful of insurance companies which offer protection against damage to the drone and personal accident cover to the operator and third-party liability exposure. Although, the entire process of documentation and securing insurance is currently cumbersome.

### 4.4. Weather-related challenges

#### 4.4.1. Differing weather conditions and wind speed at take-off and landing point and during the flight

**Figure 3:**
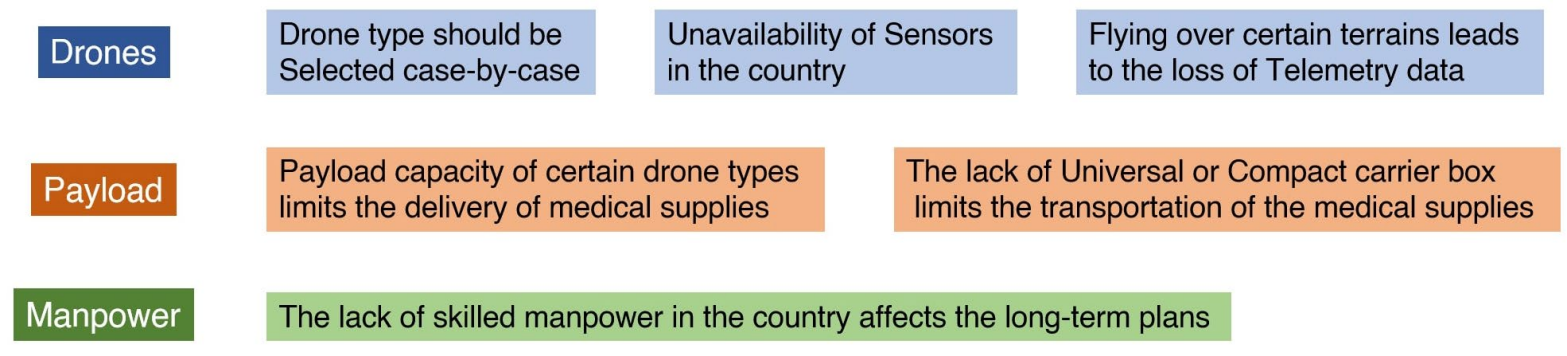
Weather related parameters which were considered prior the drone flights.

Air temperature, wind speed, precipitation, and other atmospheric phenomena have been shown to adversely affect drone endurance, control, aerodynamics, airframe integrity, line-of-sight visibility, airspace monitoring, and sensors for navigation and collision avoidance. In considering this, many drone manufacturers specify safe operating limits or warnings according to weather parameters in their manuals. In the present study, 80 sorties were conducted in both Manipur and Nagaland. However, the drone operation between the District Hospital, Bishnupur, and Primary Health Centre, Sekmaijin, Manipur was badly affected by the bad weather conditions, i.e., high wind speed and drizzling. To handle this situation, the drone was forced to make emergency landing on the transient point. This suggests that the optimal condition for flying drones would fall under the following weather limitations for the common drones, i.e., wind speed of 10 to 15 m/s and precipitation threshold of 0 to 1 mm/h (Figure 3) (21). Development of higher resolution weather forecasting technology and waterproof drones would be required in future to support drone operations in unfavourable weather conditions.

Several environmental parameters such as the altitude above sea level, relative air density, presence of high-level electromagnetic vibrations may affect the aeronautical lift which have a direct effect on the drone’s endurance. To overcome these issues, researchers have developed a risk-based framework to assess weather-related risks for local drone operations (22).

#### 4.4.2. Issues of daylight during watch hours in North-East India

Acclimatisation posed to be a foremost challenge among working teams as there were frequent weather fluctuations and the north-east region experiences early sunsets as well. Thus, fewer hours of daylight hampered the frequency of drone sorties, as the permissions were provided to only fly during daylight under the watch hours of the ATC, Imphal, Manipur from 8.15 AM till 5 PM. After initial days of observations and trials, a proper working schedule for all teams was prepared. Resultantly, the operations used to start early in the morning and end before sunset to enhance the productivity of the field operations. In this way, the staff in health care centres was also available to participate in the field operations.

### 4.5. Logistics and terrain-related challenges

#### 4.5.1. Logistics of drones, batteries, and other components to the difficult terrain

Althogh drone is a well-known machine with a wider applications, it is still associated with the sensitive concerns especially in airports, railway stations etc. Therefore, transportation of the drone and its components from the manufacturing site or warehouse to the operational site is a herculean challenge owing to the permissions required from various agencies. The lithium-ion batteries are the powerhouse for non-fuel-based drone types. These rechargeable batteries are classified based on Watt-hour rating and quantity of cells and batteries. Currently, in India, Lithium-ion batteries of watt-hour (Wh) rating above 100 Wh but less than 160 Wh are only permitted for air transportation. Hence, alternate route, i.e., train or road should be considered for the high-capacity batteries, though it is time consuming, required additional manpower and expensive. Therefore, the drone and its components should be transported with appropriate permissions from the concerned authority and as per safety protocols or SOPs for safe transportation. In the present study, the drone and its components were transported from Bengaluru, Karnataka to Imphal, Manipur through road. The transportation team was provided with all the relevant documents for the hassle-free travel through the way. It took 5 days to receive the drone and its components in Imphal. Furthermore, the drones were then transported to Imphal, Manipal to Mokokchung, Nagaland after carrying out the maintenance through road.

#### 4.5.2. Difficulties in drone operations due to the local terrain

In the present study, drone operations were conducted in various terrains including water bodies, mountains, and valleys to assess the feasibility of drones in delivering medical supplies across the terrains. The elevation of mountains in these regions necessitated development of sortie route maps and KML files from over the mountains. For this, the reliable information on the altitudes of the peaks was taken from local ATC. This information helped in planning the drone route especially between DH in Mokokchung, Nagaland to DH in Tuensang, Nagaland to achieve the maximum elevation of 6,950 feet. However, achieving high altitudes leads to increased consumption of battery, therefore to avoid any mishappening, the team plotted an alternate route with waypoints on Auto-Pilot software^©^ wherein the drone traversed between the two mountains at the lowest height.

#### 4.5.3. Unavailability of a feasible take-off and landing site in remote locations

Safe take-off and landing are essential for the completion of any drone operation. The requirements for identifying the drone take-off and landing site are, a flatland with a smooth surface not surrounded by tall objects (buildings, trees, towers) that hinders the drone flight, and satellite connectivity, restricted human movements, away from residential areas, near to the health centres etc. In the present study, such sites were identified with the help of state health authorities, and drone operators, although it was very challenging. In addition, necessary permission were accorded to the team by the district administration.

Moreover, the team members were coordinating with the district health authorities, administration, police department and the local communities for the proper administration of the drone operations in remote and interior locations. For instance, in Nagaland, the terrain posed a challenge in identifying a flat land near the health care facility for take-off and landing. Thus, a rooftop of an empty residential house adjacent to PHC premises in Ungma village, Mokokchung district, Nagaland, was identified for the landing of a quadcopter drone. The house owner was briefly explained regarding the study and he permitted the use of the roof for this operation. Therefore, in future, the unavailability of proper take-off and landing spots can be overcome by identifying such spots near to the healthcare centre, however, appropriate SOPs should be developed and approved by the concerned authorities. In addition, to enhance the precise landing of drone at such spots, an auto landing with a QR Code scanning may be facilitated.

#### 4.5.4. Lack of proper road navigation hampering accessibility to certain areas

For establishing rapport, the research team visited all 20 healthcare centres to meet the chief medical officers and other staff to explain the purpose of the study and train them for the drone-based delivery of medical supplies. Owing to the lack of metalled roads and poor internet connectivity, reaching most of these sites was challenging, however, it was an essential part to also assess the time efficacy of drones over the road transportation. For instance, in mountain terrains of Nagaland, it took around 3–4 h to cover the road distance of just 50 km, which made it difficult to conduct the drone operation on the same day. However, the aerial distance was reduced to 18 km and it took just 20 min to cover the distance. Similarly, in Manipur, the PHC Karang was located on an island which required a travel time of 2–2.5 h to cover 35 km of road distance, but via drone the aerial distance was 12 kms and it was covered in 12 min. Generally, for navigating the locations, google map was used by the team. However, there was no accessible road route from PHC Longsa to the district health centre, Mokokchung, Nagaland on google map system. This challenge was resolved by hiring a local driver who was aware of the route to Longsa village. In such cases, the drone function of automatic take-off and landing proved to be very significant to conduct operations timely.

### 4.6. Administrative issues in delivering medical supplies

Delivery of medical supplies has been decentralised in India with each state undertaking the distribution to its respective districts. PHC and CHC are connected to their district hospital or the district drug warehouse to ensure an uninterrupted medical supply. Therefore, inter-district delivery of medical supplies is not possible as each district has a limited allocation restricted for internal usage. For instance, in the present study, the longest flight covering an aerial distance of 40 Km delivered medical supplies from Mokokchung district to Tuensang district of Nagaland. However, procurement of supplies from the district drug warehouse in Mokokchung was a big obstacle due to the lack of indentation for inter-district medical deliveries in India.

### 4.7. Challenges related to social-behavioural factors

#### 4.7.1. Coordination with the local group leaders and community people

Community outreach was a tricky task due to the language barrier and cultural practices (attire, food habits, non-native) during this study at both the states. Understanding the social-behavioural factors among the stakeholders and beneficiaries was one of the major objectives to assess the acceptability of drone technology. Even though the vaccination rates in Manipur and Nagaland was satisfactory, the medical officers at a few remote locations such as P. Sajol (Churachandpur district, Manipur), Mangkolemba and Alongkima (Mokokchung, Nagaland) reported concerns about the reluctant behaviour of the smaller hamlets which restricted the entry of any healthcare official. The natives of these hamlets were frightened that the healthcare workers would spread the COVID-19 virus in their village. This kind of stigma was reported due to unawareness, fear and retaliation.

To make the locals understand the study and its possible benefits, coordination with local group leaders, youth, volunteer and self-help groups along with community people were done and rapport was established. Several awareness campaigns, vaccination drives and house-to-house visits were organised by the health centres. This helped in having effective communication with the masses in the village which was a vital step towards community outreach for achieving the study objectives. The team members also conducted various information and communication dissemination sessions with the villagers to resolve their queries and apprehension regarding drones. The locals helped in identifying the key informants for the assessment of the adaptability and acceptability of the program in the community. Moreover, they acted as useful resource persons who helped in identifying safe take-off and landing sites in the vicinity of the PHC/CHCs.

An incident which showcased the zeal of local communities towards the implementation of novel technology for the welfare of their area is as follows:

A day before the main event of transportation of vaccines from DH, Bishnupur to Karang Island, Manipur, there was a small gathering owing to the celebration of *Gandhi Jayanti* (Birthday of Mahatma Gandhi). The event was taking place at the designated landing area, due to which the drone operation was delayed. The field teams discussed this issue with the local community leaders and they agreed to cut short the duration of the event and clear the space for the proper landing of the drone thereafter. The coordination and cooperation of the local village members were commendable as they helped in warding off certain challenges which may have been difficult for the team members to address single-handedly.

#### 4.7.2. Challenge regarding language barrier

Language reportedly posed a barrier during this study in Manipur and Nagaland as most of the materials that was developed for the study was available either in English or Hindi. Thus, the native population remain at risk of being alienated from getting the necessary information about the drone-based delivery of medical supplies. To minimise the effect of the language barrier, the participant information sheet (PIS) and consent forms were translated beforehand into the local language for a better understanding of the participants. Additionally, in each district, at least two local people were appointed as field workers to facilitate communication in the absence of the health care workers or community representatives. During the in-depth interviews, each question was translated into the local language, if the respondent reported difficulty in understanding parts of it.

#### 4.7.3. Hesitation among healthcare workers involved in the study

Initially, few staff members reported their apprehensions about participation in the study due to overburden of their regular duties, reluctance towards travelling to other healthcare centres for training and inability to understand the technicalities of drone technology. This made it difficult for the research team to identify the healthcare staffs and subsequently training them. However, after deliberations and discussions, it was decided that staff members who are already working in the UIP and community members engaged in relief and voluntary work in the villages may be included in the training cohort. This was essential in terms of the capacity building since they already had the knowledge of some aspects and it was relatively easy to train them considering the time constraints.

Subsequently, the healthcare workers provided time slots for their preferred day on which the officials would visit them for training purposes. Then, once the officials were trained their interest in drone-based delivery of medical supplies grew which led to a reduction in reluctance subsequently increasing their participation in the activity.

It was made sure that the training module and presentations were prepared in easy language and visuals. In case local translations were needed, the field investigator facilitated the training session with a medical expert from the local region. Additionally, the training sessions were scheduled as per the availability of the staff. After the training program, many staff members reported that prior apprehension about the technology was alleviated, after gaining knowledge about it they feel it will be a very useful resource for emergencies.

### 4.8. Occurrence of unforeseen events

Several unforeseen events can occur while flying drones at BVLOS range such as loss of flying drone due to technical failure, sudden adverse weather conditions during mid-flight, collision with unknown objects etc. This could lead to damage to properties, human life, environment, livelihoods etc. The following steps shall be followed during unforeseen events:

- The location of the mishap should be identified through the telemetry data and the local police, district administration and community people to be informed. As their help can be useful in the identification of the spot and recovery of the drone.
- The rescue team should immediately reach the probable spot and meet the local administration and people. The team should be split into various small teams for identifying the drone.
- Also, the drone team needs to be careful and watchful of news related to the local political and social stability/Riots/ unrest etc.

While conducting the study in Nagaland, there was a clash between the civilians and military troops which led to anti-militancy operations and triggered a state-wide outrage (23). Considering the safety of the research team, drone operator and the drone, the state and district health administration requested to conclude all drone activities until the situation attained normalcy.

## 5. Discussion

The present study documented the operational challenges of the research team engaged in implementation of drone-based delivery of medical supplies in difficult terrains of North-east India. Hereby, the challenges which occurred during the planning phase, pre-flight, during flight, post-flight and during community engagement and training of healthcare workers for the drone operators were elaborately presented. Literature indicates that deployment of drones in different geographical locations often lead to site and user-specific methodological challenges which need to be bought into light for effective mitigation of the potential conflicts associated with drones (24).

The approvals of regulatory bodies, ethical committees and local stakeholders for the operations was the key priority for which variety of paper work and documentations were presented. Similar to the experience of the field team for seeking approvals for such operations in BVLOS conditions, other literary evidence also indicate that the stringent regulations and guidelines for flying drones are a common barrier for initiating drone-based healthcare deliveries (7, 25). Additionally, keeping the local ATC along with the army informed regarding the drone activities through circulation of a NOTAM was essentially used in our study, similar to drone-based operations conducted in United Kingdom (26).

Further, selection of drones, battery related issues and storage of these machineries was a technical challenge in the field for the research team as well as drone operators. In the present study, the drones were chosen on the basis of the consignment to be delivered, area of landing and take-off and the topography between the aerial route. Based on the similar criteria for selection of drone, Williams et al. (26) utilised multi-rotors instead of fixed wing gliders for stable landing and also used landing pads which led to reduce generation of dust clouds and noise.

Factors such as humidity, wind speed, air density also hinder the operations as they affect quality of sensors, camera lens, and battery (27). Similar to the experiences in this study, these weather conditions have posed challenges for drones leading to deviations from the predetermined routes (28, 29). Checking the weather prior to the operations is a pre-requisite (24). Additionally, the drone operations were restricted to watch hours of the local ATC in the present study, which is a common practice across the globe for limiting the cases of mis-happenings and accidents in night. Moreover, the noise and the light indicators of the drones may appear to be as unidentified objects in absence of proper light leading to fear among the communities. Thus, commercial operations are always allowed in watch hours and the drones are limited to fly over residential places based on their size, flying altitude and speed. Further, literature has compared historical weather data and four drone platforms to determine the percentage of the year when drone operations were possible in two different cities, which can substantially impact drone operations and limit flights to between 53.9 and 95.8% of days of the year (22). This indicates that considering the weather conditions prior to planning of drone flights is a significant step to be adopted by drone operators.

Mitigating the regulatory, environmental and weather-related challenges is not sufficient in an effective healthcare delivery mechanism using novel technology. As observed in the present study, reluctance towards drones was initially seen among the communities and stakeholders, but, interactions and awareness on the potential benefits proved significant. Similarly, use of drones has been seen to have a positive influence on local communities as they feel that it navigates in monitoring their resources efficiently (30). Duffy et al. (24) also investigated the social and ethical concerns of the engaged officials and locals similar to the approach used in this study. The social challenges associated with use of drones identified in this study were similar to the ones identified previously from literature (25, 31, 32). Additionally, collaborating stakeholders in the operation team for addressing the ethical complexities and field challenges as in the present study was priorly reported in another study in African subcontinent as well (33, 34). Thus, community engagement is an ethical and practical approach for successful implementation of any novel technology (25, 35, 36). The need for involving communities has been recognised as a vital step for conducting drone-based healthcare deliveries in several other studies similar to the observations in this study (37, 38).

A close understanding of the operational challenges faced by the research team and mitigation strategies indicate the teams’ preparedness for worst case scenario in the first experience of implementing such a novel technology in real-time at ground level. Defining the potential benefits, comprehending the occurrence of unforeseen events and steps to be taken for involvement and engagement of local stakeholders and beneficiaries has helped the team in conducting this research efficiently.

## 6. Conclusion

The team described that even in the difficult conditions, the experiences have been quite positive and the communities have gradually adopted the advent of this technology in their area. The assigned team members were able to conduct the study at all the pre-decided sites and additional locations efficiently on the suggestions of the state and district stakeholders. The acceptability of medical officials in all the locations and active participation of the healthcare staff showcased their adaptability towards this novel technology. This indicated that implementing drone based technology for delivery of medical supplies, vaccines and other relief materials will be useful in long-run in such difficult terrains which are facing delayed healthcare responses due to inaccessibility. The local communities were immensely supportive towards the officials and were delighted to witness this initiative. The project has been successful in gathering support from the communities and the stakeholders from all the locations. This descriptive paper highlighting the process of implementation of drone technology in hard-to-reach terrains, the self-experiences of the officials engaged in the project and the adaptive strategies to mitigate challenges. These experiences can be utilized by state collaborators for conducting transportation through new technology along with conventional modes in inaccessible areas as a time-efficient alternative. Conclusively, the research team looked into the concerns raised by the stakeholders and communities very closely to facilitate the drone operations in remote areas. The major lesson learnt by the team was coordination and communication with the communities and be ethically and practically correct while conducting this project.

## Data availability statement

The raw data supporting the conclusions of this article will be made available by the authors, without undue reservation.

## Author contributions

BB and SP put forth the research ideas for conducting the research project. SA and PG conceptualised the project proposal and framework for the research article. NM and SB combed the literature and drafted the article based on the experiences of the whole field team consisting of SA, PG, NM, and SB. The critical revision of the full text was done by SA and SP. SA, PG, NM, and SB contributed equally to the conceptualisation and manuscript writing. All authors contributed to the article and approved the submitted version.

## Funding

The funding for implementation of the drone-based delivery of medical supplies in North-East India was supported by Indian Council of Medical Research.

## Conflict of interest

The authors declare that the research was conducted in the absence of any commercial or financial relationships that could be construed as a potential conflict of interest.

## Publisher’s note

All claims expressed in this article are solely those of the authors and do not necessarily represent those of their affiliated organizations, or those of the publisher, the editors and the reviewers. Any product that may be evaluated in this article, or claim that may be made by its manufacturer, is not guaranteed or endorsed by the publisher.

## References

[1] Khandagale P Eranjikal D Parab S Garg B. Design and implementation of drone in healthcare applications. In ITM Web of Conferences 2021 (Vol. 40, p. 02004). EDP Sciences. doi: 10.1051/itmconf/20214002004

[2] Balasingam M. Drones in medicine—the rise of the machines. Int J Clin Pract. (2017) 71:e12989. doi: 10.1111/ijcp.12989, PMID: 28851081

[3] Meier P Soesilo D. Using drones for medical payload delivery in Papua New Guinea. Drones Human Action Case Study. (2015):2. https://drones.fsd.ch/wp-content/uploads/2016/04/Case-Study-No2-PapuaNewGuinea.pdf

[4] Reuters. Drones could speed up HIV tests in remote areas. Reuters health news. (2016). Available at: http://in.reuters.com/article/us-malawi-hiv-drones-idINKCN0XH1ZN (Accessed April 12, 2023).

[5] Srinivasan. (2020). Available at: https://niti.gov.in/planningcommission.gov.in/docs/reports/genrep/bkpap2020/26_bg2020.pdf (Accessed August 3, 2022).

[6] Chokshi M Patil B Khanna R Neogi SB Sharma J Paul VK. Health systems in India. J Perinatol. (2016) 36:S9–S12. doi: 10.1038/jp.2016.184, PMID: 27924110 PMC5144115

[7] Sarwal R Prasad U Gopal KM Kalal S Kaur D Kumar A. Investment opportunities in India's healthcare sector. (2021)

[8] Renu N. Determination of technological advancement in the era of COVID-19. New Front Med Med Res. (2021) 16:111–7. doi: 10.9734/bpi/nfmmr/v16/13205D PMC7958161 33786181

[9] Peckham R Sinha R. An architectures of health: futures for the biomedical drone. Glob Public Health. (2019) 14:1204–19. doi: 10.1080/17441692.2018.1546335, PMID: 30433846

[10] Comtet HE Johannessen KA. A socio-analytical approach to the integration of drones into health care systems. Information. (2022) 13:62. doi: 10.3390/info13020062

[11] KPMG. India’s emerging drone industry. (2023). Available at: https://kpmg.com/in/en/home/insights/2022/07/indias-emerging-drone-industry.html (Accessed March 14, 2023).

[12] Mills P.. Drone disruption: The stakes, the players, and the opportunities. Forbes. (2016). Available at: https://www.forbes.com/sites/markpmills/2016/03/23/drone-disruption-the-stakesthe-players-and-the-opportunities/#ed8a2fe7d0b5 (Accessed March 10, 2023).

[13] Chamata J Winterton J. A conceptual framework for the acceptance of drones. Int Technol Manag Rev. (2018) 7:34–46. doi: 10.2991/itmr.7.1.4

[14] Bettman JR. Perceived risk and its components: a model and empirical test. J Mark Res. (1973) 10:184–90. doi: 10.1177/002224377301000209

[15] Fischhoff B Slovic P Lichtenstein S Read S Combs B. How safe is safe enough? A psychometric study of attitudes towards technological risks and benefits. Policy Sci. (1978) 9:127–52. doi: 10.1007/BF00143739

[16] Ministry of Civil Aviation. New drone Rules-2021. Press Information Bureau. (2022). Available at: https://static.pib.gov.in/WriteReadData/specificdocs/documents/2022/jan/doc202212810701.pdf (Accessed August 3, 2022).

[17] Airports Authority of India. What is a NOTAM? (2022). Available at: https://www.aai.aero/en/content/what-notam (Accessed August 3, 2022).

[18] Peri D Lalithangbam I. Assam rifles commanding officer, family, four jawans killed in Manipur ambush. The Hindu. (2021). Available at: https://www.thehindu.com/news/national/other-states/assam-rifles-co-others-killed-inmanipur-ambush/article37469920.ece (Accessed December 27, 2021).

[19] Andújar D Rueda-Ayala V Moreno H Rosell-Polo JR Escolá A Valero C. Discriminating crop, weeds and soil surface with a terrestrial LIDAR sensor. Sensors. (2013) 13:14662–75. doi: 10.3390/s131114662, PMID: 24172283 PMC3871132

[20] Townsend A Jiya IN Martinson C Bessarabov D Gouws R. A comprehensive review of energy sources for unmanned aerial vehicles, their shortfalls and opportunities for improvements. Heliyon. (2020) 6:e05285. doi: 10.1016/j.heliyon.2020.e05285, PMID: 33235928 PMC7672221

[21] Gao M Hugenholtz CH Fox TA Kucharczyk M Barchyn TE Nesbit PR. Weather constraints on global drone flyability. Sci Rep. (2021) 11:12092. doi: 10.1038/s41598-021-91325-w, PMID: 34103585 PMC8187708

[22] Roseman CA Argrow BM. Weather hazard risk quantification for sUAS safety risk management. J Atmos Ocean Technol. (2020) 37:1251–68. doi: 10.1175/JTECH-D-20-0009.1

[23] Yhoshü A.. Nagaland’s Mon district tense after stor over civilian killings turn violent. India News. (2021). Available at: https://www.hindustantimes.com/indianews/nagalands-mon-district-tense-after-stir-over-civilian-killings-turns-violent101638730295882.html (Accessed December 29, 2021).

[24] Duffy JP Cunliffe AM DeBell L Sandbrook C Wich SA Shutler JD. Location, location, location: considerations when using lightweight drones in challenging environments. Remote Sens Ecol Conserv. (2018) 4:7–19. doi: 10.1002/rse2.58

[25] Jeyabalan V Nouvet E Meier P Donelle L. Context-specific challenges, opportunities, and ethics of drones for healthcare delivery in the eyes of program managers and field staff: a multi-site qualitative study. Drones. (2020) 4:44. doi: 10.3390/drones4030044

[26] Williams GD Fraser AD Lucieer A Turner D Cougnon E Kimball P. Drones in a cold climate. Eos. (2016) 97:1–6. doi: 10.1029/2016EO043673

[27] Wadcock AJ Ewing LA Solis E Potsdam M Rajagopalan G. Rotorcraft downwash flow field study to understand the aerodynamics of helicopter brownout. California, United States of America: National Aeronautics and Space Administration Moffett Field Ca Ames Research Center (2008).

[28] Jordan BR. A birds-eye view of geology: the use of micro drones/UAVs in geologic fieldwork and education. GSA Today. (2015) 25:50–2. doi: 10.1130/GSATG232GW.1

[29] Cunliffe AM Anderson K DeBell L Duffy JP. A UK civil aviation authority (CAA)-approved operations manual for safe deployment of lightweight drones in research. Int J Remote Sens. (2017) 38:2737–44. doi: 10.1080/01431161.2017.1286059

[30] Paneque-Gálvez J McCall MK Napoletano BM Wich SA Koh LP. Small drones for community-based forest monitoring: an assessment of their feasibility and potential in tropical areas. Forests. (2014) 5:1481–507. doi: 10.3390/f5061481

[31] Sandbrook C. The social implications of using drones for biodiversity conservation. Ambio. (2015) 44:636–47. doi: 10.1007/s13280-015-0714-0, PMID: 26508350 PMC4623858

[32] Boucher P. Domesticating the drone: the demilitarisation of unmanned aircraft for civil markets. Sci Eng Ethics. (2015) 21:1393–412. doi: 10.1007/s11948-014-9603-3, PMID: 25371277 PMC4656702

[33] Kuponiyi FA. Community power structure: the role of local leaders in community development decision making in Ajaawa, Oyo state. Nigeria Anthropol. (2008) 10:239–43. doi: 10.1080/09720073.2008.11891056

[34] Brear M. Ethical research practice or undue influence? Symbolic power in community-and individual-level informed consent processes in community-based participatory research in Swaziland. J Empir Res Hum Res Ethics. (2018) 13:311–22. doi: 10.1177/1556264618761268 29529932

[35] Gomez R Reed P Chae HY. Assessment of community wellness outcomes to measure ICT impact. In Proceedings of the Sixth International Conference on Information and Communications Technologies and Development: Notes-Volume 2 (2013) (pp. 37–40).

[36] Resnik DB Elliott KC. Using drones to study human beings: ethical and regulatory issues. Sci Eng Ethics. (2019) 25:707–18. doi: 10.1007/s11948-018-0032-6, PMID: 29488061 PMC6111004

[37] Geoffroy E Harries AD Bissell K Schell E Bvumbwe A Tayler-Smith K. Bringing care to the community: expanding access to health care in rural Malawi through mobile health clinics. Public Health Action. (2014) 4:252–8. doi: 10.5588/pha.14.0064, PMID: 26400705 PMC4533507

[38] Sham R Siau CS Tan S Kiu DC Sabhi H Thew HZ. Drone usage for medicine and vaccine delivery during the COVID-19 pandemic: attitude of health care workers in rural medical centres. Drones. (2022) 6:109. doi: 10.3390/drones6050109

